# Promises of Big Data and Artificial Intelligence in Nephrology and Transplantation

**DOI:** 10.3390/jcm9041107

**Published:** 2020-04-13

**Authors:** Charat Thongprayoon, Wisit Kaewput, Karthik Kovvuru, Panupong Hansrivijit, Swetha R. Kanduri, Tarun Bathini, Api Chewcharat, Napat Leeaphorn, Maria L. Gonzalez-Suarez, Wisit Cheungpasitporn

**Affiliations:** 1 Division of Nephrology, Department of Medicine, Mayo Clinic, Rochester, MN 55905, USA; charat.thongprayoon@gmail.com (C.T.); api.che@hotmail.com (A.C.); 2 Department of Military and Community Medicine, Phramongkutklao College of Medicine, Bangkok 10400, Thailand; wisitnephro@gmail.com; 3 Division of Nephrology, Department of Medicine, University of Mississippi Medical Center, Jackson, MS 39216, USA; kkovvuru@umc.edu (K.K.); skanduri@umc.edu (S.R.K.); mgonzalezsuarez@umc.edu (M.L.G.-S.); 4 Department of Internal Medicine, University of Pittsburgh Medical Center Pinnacle, Harrisburg, PA 17105, USA; hansrivijitp@upmc.edu; 5 Department of Internal Medicine, University of Arizona, Tucson, AZ 85721, USA; tarunjacobb@gmail.com; 6 Department of Nephrology, Department of Medicine, Saint Luke’s Health System, Kansas City, MO 64111, USA; napat.leeaphorn@gmail.com

**Keywords:** artificial intelligence, machine learning, big data, nephrology, transplantation, kidney transplantation, acute kidney injury, chronic kidney disease

## Abstract

Kidney diseases form part of the major health burdens experienced all over the world. Kidney diseases are linked to high economic burden, deaths, and morbidity rates. The great importance of collecting a large quantity of health-related data among human cohorts, what scholars refer to as “big data”, has increasingly been identified, with the establishment of a large group of cohorts and the usage of electronic health records (EHRs) in nephrology and transplantation. These data are valuable, and can potentially be utilized by researchers to advance knowledge in the field. Furthermore, progress in big data is stimulating the flourishing of artificial intelligence (AI), which is an excellent tool for handling, and subsequently processing, a great amount of data and may be applied to highlight more information on the effectiveness of medicine in kidney-related complications for the purpose of more precise phenotype and outcome prediction. In this article, we discuss the advances and challenges in big data, the use of EHRs and AI, with great emphasis on the usage of nephrology and transplantation.

## 1. Introduction

Kidney diseases, such as acute kidney injury (AKI) and chronic kidney disease (CKD) are major medical and public health issues worldwide, associated with high death and morbidity rates, together with great economic loss [1,2,3,4,5,6]. CKD is linked with a higher danger of argumentative outcomes, like cardiovascular complications, death, decreased quality of life, and substantial healthcare resource utilization [7,8,9,10,11], and it has been assessed that around 850 million individuals suffer different types of kidney diseases globally [12,13]. If left untreated, CKD may evolve into end-stage kidney disease (ESKD), which is associated with high mortality [14,15,16]. It is well-known that kidney diseases are very much multifactorial, with overlapping and complex clinical phenotypes, as well as morphologies [17]. The global distribution of nephrologists usually differs from one country to another, with bigger differences in its overall capacity [18]. Various nations across the world have established surveillance systems for kidney-related infections. Despite such attempts, the literature highlights that surveillance systems within third world countries are still not very strong [19]. In certain areas of some countries, basic records offices for transplantation and dialysis, as well as expert pathologists, are not even available [18,20]. Given how major gaps are always found in the main workforce in nephrology, the current eminence of kidney health management and research evidence in nephrology needs to be strengthened globally [21]. 

Traditionally, the randomized controlled trial (RCT) has always been used as the point of reference for offering evidence-based treatment. The numerical formulae applied in analyzing randomized control data have equally offered essential insights from numerous observational data. In the past few years, great emphasis has been placed on the pragmatic RCT, an essential component of real global research, which is applied when evaluating the great interventions within the actual clinical setting based on a great amount of samples so as to stimulate their individual practical value. A great amount of differences have been reported within nephrology, as well as some other relevant specialties. For instance, the literature indicates that nephrology trials were very limited in number and possessed minimally optimal features of high-quality designs [22]. Despite the fact that the already existing studies, as well as implemented works, have made major additions to a highly reliable prognostication, as well as an extensive understanding of the general histologic pathology, there is still a great amount of work which needs to be undertaken, as well as specific problems to be solved. The general capacity for undertaking cohort studies that involve a large sample size or Rapid Control trial is very much present across various parts of the globe, and has thereby resulted in the absence of research evidence within nephrology. In addition, limited activity in kidney research has impacted the evidence base for the treatment of kidney diseases, resulting in a lack of useful surrogate end-points for progression from the early stages of kidney disease-hindered trials [14,15]. On the same note, a great amount of cohort data could also be applied in generating relevant hypotheses and provide major insights into the etiology, pathogenesis, and prognosis of kidney diseases [23,24].

Those needs that are classified as unmet require provision of some ample spaces for the purpose of imagination in relation to leveraging the strength associated with big data, as well as relevant artificial intelligence (AI) to improve the overall status of patients with kidney diseases [25]. In this article, we discuss the big data concepts in nephrology, describe the potential use of AI in nephrology and transplantation, and also encourage researchers and clinicians to submit their invaluable research, including original clinical research studies [26,27,28,29,30], database studies from registries [31,32,33], meta-analyses [34,35,36,37,38,39,40,41,42,43,44], and artificial intelligence research [25,45,46,47,48] in nephrology and transplantation. 

## 2. Big Data in Nephrology and Transplantation: Registries and Administrative Claims

Table 1 demonstrates known and commonly used databases that have provided big data in nephrology and transplantation [49,50,51]. For example, the United States Renal Data System (USRDS) is recognized as a state reconnaissance system that has the responsibility of collecting, analyzing, and subsequently distributing information regarding CKD and ESKD, all based on numerous big datasets. By delivering the yearly data report, the USRDS continuously tracks both the epidemiologic and economic burden linked to kidney diseases [52]. An important database in transplantation in the United States is the United Network for Organ Sharing (UNOS). The Organ Procurement and Transplantation Network (OPTN) data are linked by UNOS to the Social Security Death Master File for the purpose of augmenting ascertainment of different groups of candidates, as well as relevant deaths. The final data are attainable by different groups of researchers, and have always been applied in various studies regarding transplantation [50]. In addition to these databases in the United States, other countries worldwide also have big data within nephrology for researchers, such as the National Kidney Disease Surveillance Program in Ireland [53], the surveillance project on CKD management in Canada [54], and the China Kidney Disease Network (CK-NET), a comprehensive CKD surveillance system for China [55]. 

Numerous networks of international collaboration, like the International Network of CKD cohorts [56], the Therapeutic Evaluation of Steroids in IgA Nephropathy Global study [57], and the Chronic Kidney Disease Prognosis Consortium [58] have grown immensely within the last few years. There are possible advantages of introducing a traditional data element that are linked to kidney infections, like escalating the overall power of the groups which are under-represented [59]. There is, however, great need to address numerous challenges, like standardization of data, identification of the patient, plus some other additional infrastructure-related challenges. Additionally, the cadre of genomics is developing at a very rapid rate towards realizing an analysis of single cells, and subsequent great advances within metabolomics and proteomics have been developed within the past few years. A great amount of progress has equally been realized within technological developments within the areas of large-scale molecular data generation in various databases that are gene-based (Table 1). The most recent advancements in technology have made it possible for us to produce larger amounts of data, more specifically regarding the omics data. Further development of somehow less expensive genotype arrays and the subsequent presence of samples within biobanks made it possible to undertake genome-wide association studies among numerous groups of patients, offering highly essential insights into the great risk factors and the pathogenesis of multiple kidney diseases [60,61,62,63]. 

Within nephrology, numerous consortia-collecting biopsy biobanks of kidney tissue have been started to undertake such forms of collaborative study. Several initiatives that are aimed at extensive characterization of the relevant kidney biopsies for various groups of kidney diseases subtypes have subsequently been launched, comprising of the NEPTUNE (Nephrotic Syndrome STudy Network), ERCB (European Renal cDNA Bank), EURenOmics, C-PROBE (Clinical Phenotyping and Resource Biobank), PKU-IgAN, and more recently, TRIDENT (for diabetic nephropathy), CureGN (for glomerulopathies), and the NIDDK (National Institute of Diabetes and Digestive and Kidney Diseases) Kidney Precision Medicine Project (KPMP) [64].

Big data within the medicine field might offer the opportunity to envision patients suffering from kidney diseases in a more holistic manner, using numerous lenses, each of which adequately presents the great opportunity of studying various scientific queries. Such data within the big databases might subsequently comprise of the general administrative health-related data, biometric data, biomarker data, as well as imaging, and might subsequently come from various sources, comprising of electronic health records, biobanks, reports in the internet, and various clinical registries [65]. These data from the large databases are collected and updated overtime. These data are valuable and can be used by researchers to answer numerous research questions and advance knowledge in nephrology and transplantation [66,67,68].

## 3. Using Electronic Health Record Data in Nephrology 

Two major events have been reported within the last 10 years that seem to have changed the whole situation. To begin with is making it possible to digitalize all relevant medical information—more specifically, the initiation of EHRs that have the medical histories of the patients—and facilitate the processing of medical information using computers. This helps to make information-processing become automated by the use of given specialized software. EHRs have been greatly utilized with major regularity, clinical informatics strategies have subsequently been refined, and subsequently, the EHR field enabled [69,70]. 

The wide application of EHRs, when put together with the relevant novel of big data, tends to create some forms of unique opportunities for the purpose of nephrology research, as well as improvement in care for individual patients who might be suffering from kidney complications and transplantation. The data which is there within the EHR is considered big insofar as its volume is concerned. Such interventions have resulted in a new era of big data which has subsequently fueled precision medicine. These types of approach have already indicated an improved level of diagnosis, risk assessment, as well as treatment and management of numerous health conditions. With medicine getting digitalized, a great amount of data has since been developed from all aspects of health care, comprising the laboratory tests, EHR, together with medical imaging. 

For instance, in the instance of electronic AKI, the automated diagnostic strategy tends to create a great opportunity to initiate predictive strategies, optimize the relevant AKI alerts, and subsequently trace AKI events across various institutions, as well as administrative datasets. The growth in the adoption of EHR and subsequent maturation of the relevant clinical informatics techniques might provide some sort of unique opportunity to advance the general predictive capabilities. Immediately, AKI has been properly diagnosed within real time, and several EHR-enabled interventions have become so viable. One of such great prospects is actually the prediction of detecting events prior to their occurrence [71]. AKI events might temporarily get anchored within the EHR, which develops a pre-disease phase of care, having the information which had accumulated before the development of AKI. With a greater amount of content, high-throughput strategies can be applied to such a group of data so as to help in identifying a form of pre-AKI signal, which can subsequently assist in discriminating between patients who are of high risk and low risk for the AKI. The capability to predict AKI risk in this manner might subsequently have some forms of dramatic impact, as presently there is no scientifically proven treatment for AKI once one develops such conditions [72]. As patients who are considered to be of high risk get identified, the extent of care can get modified, and further strategies for harm prevention implemented. In the long run, such groups of patients, institutions, and population-based techniques will result in better long- and short-term outcomes for the respective hospitals, patients, and the whole of the healthcare system. Despite the fact that potential barriers are always there, and several nuanced groups need to be taken into consideration, such approaches that are EHR-enabled have the ability to greatly improve AKI-associated knowledge and care. 

Patients suffering from kidney complications are reported to have the highest level of heterogeneity in manifestation of the disease, treatment response, and overall progression. The growth in big data actually tends to stimulate the general boom of artificial intelligence that is a perfect tool that helps in handling, and also processing the big data. AI can assist in shedding light on the specific accuracy of medicines used to treat kidney diseases, for outcome prediction, and also to gain a more precise phenotype.

## 4. Artificial Intelligence in Nephrology and Transplantation

**Figure 1:**
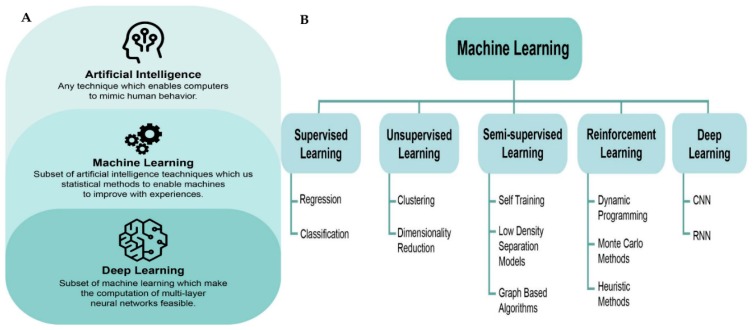
(**a**) Relationships between artificial intelligence, machine learning, and deep learning. (**b**) Types of machine learning. CNN, convolutional neural network; RNN, recurrent neural network.

AI presently shows a very important function in nearly all areas of the day-to-day lives of human beings, as well as within different academic disciplines. Based on the fact that there has been growth in the power of computers, developments in techniques and methods, and the overall blast of the quantity of medicine, data has never been an exemption. Literature clarifies that artificial intelligence can be used in disease risk assessment. Actually, disease risk assessment has a very important influence on the general prognosis, as well as clinical intervention strategies. Accurate and rapid assessment can assist clinicians in determining the conditions of the patient, out of which optimal treatment strategies can be implemented. Links between prognosis and risk factors of the diseases are very complex. The same risk factors can be experienced within different groups of diseases, and a single disease can actually be composed of several risk factors. In such case, the links between the known risk factors and the disease has very strong correlativity, instead of simple causality. Artificial intelligence can hence be applied in doing disease risk assessment in order to understand the main factors linked to disease prognosis so as to offer effective treatment for tertiary prevention of the disease. One of the important sections of AI is machine learning, which is characterized as the study of algorithms and statistical models that computer systems utilize to learn from sample data and previous experience without being explicitly programmed to achieve particular assignments. With the ability to identify obscure patterns in the data, we can use machine learning to solve many problems, including assessing relationships of two variables, creating predictions based on baseline characteristics, identifying objects with comparable patterns, and incorporating subjects by specific criteria. Machine learning techniques have the capacities of managing complex datasets and tremendous numbers of variables that are exceeding the capability of classical statistical methods [17]—see Figure 1A. Machine learning algorithms are usually utilized without initiating as many presumptions of the underlying data. In addition, a machine-learning method can determine complex patterns of health trajectories of immense numbers of characteristics and patients, which has exhibited high predictive certainty, and been confirmed and replicated with various validation investigations [73]. Well-known machine-learning algorithms include the artificial neural network (ANN), random forest, gradient boosting trees, and support vector machine [17].

Inspired by the idea of mimicking the biological structure of human brains, deep learning is a subfield of machine learning based on ANN [74]. Deep-learning models can learn many levels of data design with a multiple-processing-layers model structure, attaining more powerful model performance. This cutting-edge technology has significantly changed the paradigm of visual object recognition, speech recognition, and many other domains, such as genomics and drug discovery. Deep learning techniques are increasingly being applied to biomedical data, from image processing to genomic data analysis [75]. Such methods might outperform pathologists’ fibrosis scores from histological renal biopsy images [76]. Well-known techniques include the convolutional neural network (CNN), fully connected neural network, generative adversarial network (GAN), deep reinforcement learning, and recurrent neural network (RNN) [17,77], shown in Figure 1B. AI-based clinical decision support systems (CDSS) can be implemented employing the expert system strategy, data-driven approach, or an ensemble approach by coupling both. An expert system consolidates a knowledge base containing a set of rules for specific clinical scenarios, and the initial rule set may be acquired from domain experts or learned from data through machine learning algorithms [72,78,79,80]. 

AI has recently been adopted for the prediction, diagnosis, and treatment of kidney diseases [76,81,82,83,84,85], as shown in Table 2. For example, a prediction model based on the combination of a machine learning algorithm and survival analysis has recently developed and can stratify risk for kidney disease progression among patients with IgA Nephropathy [86]. For AKI research, Tomasev et al. [83] recently used deep-learning methods to make a continuous prediction of AKI by developing a RNN model on the sequential health record data of >700,000 veterans, allowing physicians to practise with adequate data and sufficient time. In addition, regarding utilization of ANN and CNN methods, Kolachalama et al. [76] recently provided a perspicacity into the association of pathological fibrosis identified from histologic images with clinical phenotypes for patients with CKD, helping the diagnostics and prognostics of these phenotypes. Subsequently, there has been an increasing number of AI studies, with great emphasis on the usage of nephrology and transplantation [85,87,88,89].

## 5. Potential Directions and Future Scope

In order to reinforce the usage and subsequent transformation of AI as well as data–based CDSSs in nephrology, AI, as well as big data, offers the chance to actually source knowledge from expert knowledge and big data and subsequently transform it into some form of intelligent system, which can be applied in risk classification, disease diagnosis, drug discovery, and prognostic evaluation, among some other things. AI might be useful in establishing the type of kidney disease and subsequently help in solving problems related to survival analysis of the patients who have gone through kidney transplants [106,107,108,109,110,111,112,113,114]. Renal biopsy images may be a good data base for application of machine learning algorithms.

Despite having numerous imperfections, big data, as well as artificial intelligence have been applied in the field of medication from numerous parts [115,116]. There are numerous possible guidelines of using big data and artificial intelligence in nephrology that requires greater attention, as well as further consideration [74,78,117,118,119,120,121,122,123,124,125].

## 6. Conclusions 

In summary, the present status of kidney health care, and subsequently, research evidence in nephrology requires strengthening. Big data research that is problem-driven in nephrology is very much essential in promoting the interdisciplinary incorporation and subsequent improvements in kidney disease, and it may subsequently offer some greater insights to further studies in the future. Within the present era of using big data, it is strongly believed that big data and artificial intelligence will greatly reshape research done on kidney disease and consequently improve the general clinical practice of nephrology in the near future.

## Figures and Tables

**Table 1:** Nephrology and transplant databases and organizations.

Renal and Transplant Databases	Organizations
United States Renal Data System (USRDS) (https://www.usrds.org) Organ Procurement and Transplantation Network (OPTN) (https://optn.transplant.hrsa.gov) United Network for Organ Sharing (UNOS) (https://unos.org) Scientific Registry of Transplant Recipients (SRTR) (https://www.srtr.org) National Health and Nutrition Examination Survey Database (NHANES) (https://www.cdc.gov/nchs/nhanes/index.htm) Chronic kidney disease database (CKDd) (http://www.padb.org/ckddb/) National Death Index (NDI) (https://www.cdc.gov/nchs/ndi/index.htm) Nephrotic Syndrome Study Network (NEPTUNE) (https://nephcure.org/2015/12/nephrotic-syndrome-study-network-neptune/) National Inpatient sample (NIS) (https://www.hcup-us.ahrq.gov/news/exhibit_booth/nis_brochure.jsp) Polycystic Kidney Disease Consortium Data Base (PKD) (https://pkdcure.org/research-medical-professionals/pkdoc/) Kidney Early Evaluation Program Data base (KEEP) (https://www.kidney.org/news/keep) Diabetes mellitus Treatment for Renal Insufficiency Consortium Database (DIAMETRIC) (https://www.diametric.nl/diametric-database/) Center for Medicare And Medicaid Services (CMS) (https://www.cms.gov) Jackson Heart Study (JHS) (https://www.jacksonheartstudy.org) **Gene Based Databases:** CKD- Gen Consortium Database (https://ckdgen.imbi.uni-freiburg.de) Genome Wide Association Studies (GWAS) (https://dceg.cancer.gov/research/how-we-study/genomic-studies/gwas-overview) Nephro Seq (https://www.nephroseq.org/resource/login.html) Renal gene Expression Database (http://rged.wall-eva.net) Humana Kidney and Urine Proteome Project (HKUPP) (http://www.hkupp.org) Urine protein Biomarker Database (http://122.70.220.102/biomarker) Urinary Peptidomics and Peak- maps (http://www.padb.org/updb/) Kidney and Urinary Pathway Knowledge Database (KUPKB) (http://www.kupkb.org)	ESRD Networks (https://esrdnetworks.org) American Society of Nephrology (ASN) (https://www.asn-online.org) National Kidney Foundation (NKF) (https://www.kidney.org) International Society of Nephrology (ISN) (https://www.theisn.org) American Transplant Congress (ATC) (https://atcmeeting.org) Renal Physician Association (RPA) (https://www.renalmd.org) International Society of Peritoneal Dialysis (ISPD) (https://ispd.org) National Renal Administrators Association (NRAA) (https://www.nraa.org/home) Kidney and Urology Foundation of America (http://www.kidneyurology.org) American Kidney Fund (https://www.kidneyfund.org/about-us/) American Society of Artificial Internal Organs (https://asaio.org/about/) Organ Procurement Organization (OPO) (https://unos.org/transplant/opos-increasing-organ-donation) Acute Dialysis Quality Initiative (ADQI) (http://www.adqi.net/) National Institute of Health (NIH) (https://www.nih.gov) National Institute of Diabetes and Digestive and Kidney Diseases (https://www.niddk.nih.gov) National Center for Health Statistics (NCHS) (https://www.cdc.gov/nchs/about/index.htm)

**Table 2:** Selected articles reporting the utilization of artificial intelligence, machine learning, or deep learning in the field of nephrology and kidney transplantation.

Study	Country	Study Type	N	Subjects	Intervention
Zhou, 2020 [90]	China	R	212	Prediction of ARF and paraplegia after TAAAR	Machine learning classification models
Xu, 2020 [91]	USA	R	37,486	Identification the sub-phenotypes of AKI	Memory network-based deep learning approach
Song, 2020 [92]	USA	R	14,039	Longitudinal Risk Prediction of CKD in Diabetic Patients	Temporal-enhanced gradient boosting machine
Rashidi, 2020 [93]	USA	P, R	101	Early recognition of burn- and trauma-related AKI	Artificial intelligence /machine learning algorithms
Morid, 2020 [94]	USA	R	22,542	Prediction of adverse events in critical patients with AKI	Temporal pattern detection
Luo, 2020 [95]	China	R	519	Prediction of severe pneumonia during post-transplant hospitalization in recipients of a deceased-donor kidney transplant	Machine learning
Li, 2020 [96]	China	P	1952	Accuracy improvement of GFR estimation	Artificial neural network
Lei, 2020 [97]	China	R	1173	Prediction of AKI after liver cancer resection	Machine learning algorithms
Kate, 2020 [98]	USA	R	44,691	Prediction of AKI in hospitalized patients	Machine learning predictive models
Kang, 2020 [99]	South Korea	R	1571	Prediction of mortality in CRRT patients	Machine learning algorithms
Zimmerman, 2019 [100]	USA	R	23,950	Prediction of AKI following ICU admission	Machine learning models
Zhang, 2019 [101]	China	R	2456	Prediction of volume responsiveness in oliguric AKI	Machine learning models
Xu, 2019 [102]	USA	R	58,976	Prediction of mortality in patients with AKI in the ICU	Machine learning models
Xiao, 2019 [103]	China	R	551	Prediction of CKD progression	Machine learning tools
Mark, 2019 [104]	USA	R	100,000	Prediction of survival of kidney transplant recipients from UNOS	Machine learning models
Bae, 2019 [105]	USA	R	120,818	Prediction of survival after deceased donor kidney transplant from OPTN database	Machine learning methods

AKI, acute kidney injury; ARF, acute renal failure; AUC, area under curve; CKD, chronic kidney disease; CRRT, continuous renal replacement therapy; GFR, glomerular filtration rate; ICU, intensive care unit; OPTN, Organ Procurement and Transplantation Network; P, prospective; R, retrospective; TAAAR, thoracoabdominal aortic aneurysm repair; UNOS, United Network for Organ Sharing.
